# Quality of Layperson CPR Instructions From Artificial Intelligence Voice Assistants

**DOI:** 10.1001/jamanetworkopen.2023.31205

**Published:** 2023-08-28

**Authors:** William Murk, Eric Goralnick, John S. Brownstein, Adam B. Landman

**Affiliations:** 1 Department of Emergency Medicine, Albert Einstein College of Medicine, The Bronx, New York; 2 Department of Emergency Medicine, Brigham and Women’s Hospital, Boston, Massachusetts; 3 Harvard Medical School, Boston, Massachusetts; 4 Innovation and Digital Health Accelerator, Boston Children’s Hospital, Boston, Massachusetts

## Abstract

This case series study evaluates responses from 4 artificial intelligence voice assistance on CPR questions from laypersons.

## Introduction

Despite widespread cardiopulmonary resuscitation (CPR) courses, 46% of out-of-hospital cardiac arrest cases have layperson CPR performed.^1^ Layperson CPR is associated with an increase survival by 2- to 4-fold.^2^ Artificial intelligence (AI) voice assistants (VAs) are becoming ubiquitous and may be a novel method to provide verbal CPR instructions during an emergency. These technologies are used by nearly half of US adults^3^ and are increasingly being used for health care needs.^4^ Although bystanders may obtain CPR instructions from emergency dispatchers, such services are not universally available, and their use may be limited by language barriers, poor audio quality, call disconnection, fear of law enforcement, and perceived costs.^5^ VAs may therefore serve as a source of readily accessible CPR instruction when it otherwise may be unavailable.

We aimed to evaluate 4 VAs for their out of the box ability to provide appropriate CPR instruction. We also tested a recently developed AI large language model (LLM) for its ability to provide CPR instruction. Although this LLM natively offers only text-based interaction and is therefore not a VA, we sought to evaluate its performance given that it may reflect future VA capabilities.

## Methods

This case series did not include human participants and did not meet the criteria for human participant research by the Mass General Brigham Institutional Review Board. In February 2023, we tested several ways of asking about CPR on 4 VAs (Amazon Alexa on Echo Show 5, Apple Siri on iPhone 14 Pro [iOS 16.2], Google Assistant on Nest Mini, and Microsoft Cortana on a Windows 10 [ 21H2] laptop). We asked 8 verbal queries of each VA. Transcriptions of interactions were reviewed to verify that queries were accurately captured. We also asked the queries to the LLM ChatGPT version 3.5 (OpenAI) on February 13 via text. The quality of responses was rated by 2 board-certified emergency medicine physicians (E.G. and A.B.L.).

## Results

**Figure 1.**
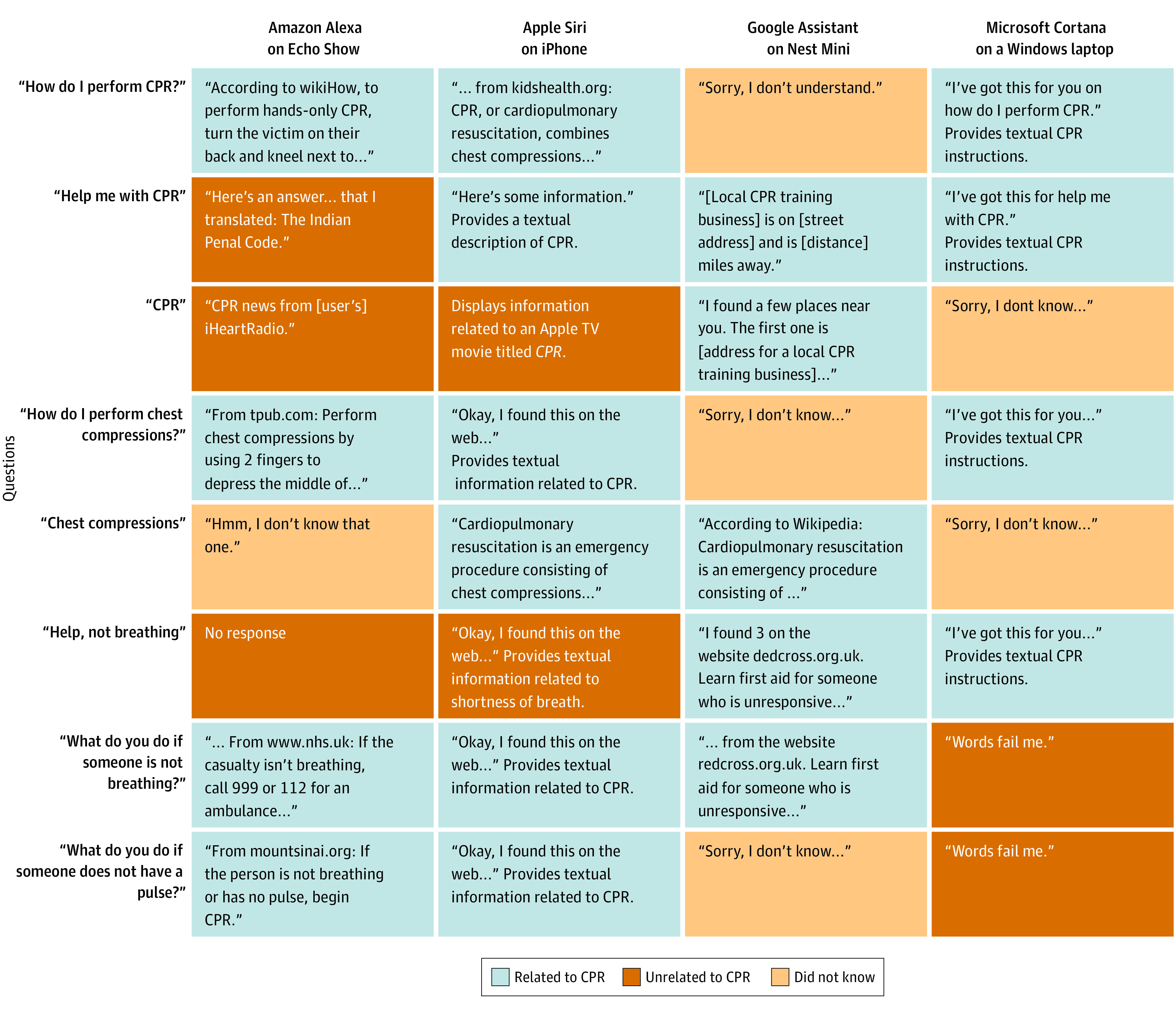
Responses to Questions Responses to cardiopulmonary resuscitation (CPR) questions by artificial intelligence voice assistants are presented, colored according to whether the response was determined to be related to CPR (providing information pertaining to CPR or recommending the use of emergency services) or unrelated to CPR or if the VA acknowledged that it did not know the answer. Responses shown here are abbreviated versions of full response transcriptions.

**Figure 2.**
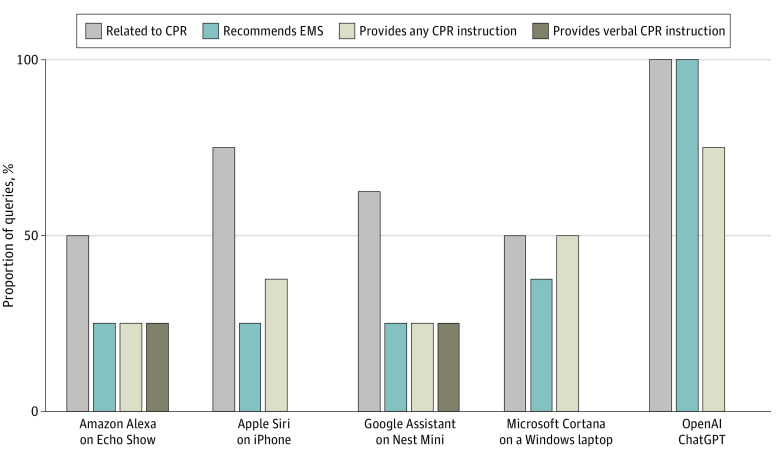
Summary of Responses Responses to 8 cardiopulmonary resuscitation (CPR) questions are presented by product. EMS indicates emergency medical services.

Figure 1 illustrates VA responses to CPR queries. Of 32 responses, 19 responses (59%) were related to CPR. There were 9 responses (28%) that suggested calling emergency services, 11 responses (34%) that provided any (verbal or textual) CPR instructions, and 4 responses (12%) that provided verbal instructions. Differences in appropriateness of responses were noted. For example, while 1 VA more frequently provided CPR instructions than other VAs, these instructions were textual only (Figure 2). The LLM provided relevant CPR information for 100% of queries and textual CPR instructions for 75% of queries (Figure 2). Among 17 responses from VAs or the LLM providing CPR instruction, 71% described hand positioning, 47% described compression depth, and 35% described compression rate.

## Discussion

This case series study found that nearly half of queries were answered by VAs with information unrelated to CPR, often constituting grossly inappropriate responses. These findings suggest that a layperson seeking to use a VA for CPR guidance may experience delays or fail to find appropriate content. For example, with only 28% of VA responses suggesting calling emergency services, use of a VA may be associated with delays in contact with medical care. Although the LLM had improved performance compared with VAs, its responses were inconsistent. Study limitations include a small number of queries and not assessing how responses changed over time.

These findings suggest that bystanders should prioritize calling emergency services over using a VA, especially given that bystanders may not recognize a patient in cardiac arrest. This recognition can be challenging even for dispatchers, and this may be another problem that can be addressed with machine learning.^6^

VAs need to better support CPR by: (1) building CPR instructions into core functionality, (2) designating common phrases to activate CPR instructions, and (3) establishing a single set of evidence-based content items across devices, including prioritizing calling emergency services for suspected cardiac arrest. The technology industry could partner with the medical community and professional societies to standardize VA support for CPR instruction.

## Notes

Supplement.
Data Sharing Statement
