# Supplementary material for: Quality of Layperson CPR Instructions From Artificial Intelligence Voice Assistants
Source: JAMA Netw Open. 2023 Aug 28;6(8):e2331205. doi: 10.1001/jamanetworkopen.2023.31205 (PMC10463098; doi:10.1001/jamanetworkopen.2023.31205)
Supplement: Supplement. — Data Sharing Statement [file jamanetwopen-e2331205-s001.pdf]

Data Sharing Statement

Murk. Quality of Layperson CPR Instructions From Artificial Intelligence Voice Assistants. JAMA Netw Open. Published online August 28, 2023. doi:10.1001/jamanetworkopen.2023.31205

Data
Data available: Yes
Data types: Data (not involving human participants) **How to access data:** All study data are available at https://doi.org/10.6084/m9.figshare.23497751.v3 . **When available:** With publication

Supporting Documents
Document types: None

Additional Information
Who can access the data: anyone requesting the data
Types of analyses: for any purpose
Mechanisms of data availability: for any purposes
Any additional restrictions: none
